# Preparation and Application of Molecularly Imprinted Monolithic Extraction Column for the Selective Microextraction of Multiple Macrolide Antibiotics from Animal Muscles

**DOI:** 10.3390/polym11071109

**Published:** 2019-07-01

**Authors:** Xuqin Song, Tong Zhou, Jiahui Zhang, Yijuan Su, Hao Zhou, Limin He

**Affiliations:** 1 National Reference Laboratory of Veterinary Drug Residues (SCAU), College of Veterinary Medicine, South China Agricultural University, Guangzhou 510642, China; 2 Department of Ecology, College of Natural Resources and Environment, South China Agricultural University, Guangzhou 510642, China

**Keywords:** molecularly imprinted monolithic extraction column, microextraction, macrolide antibiotics, animal muscles, liquid chromatography-tandem mass spectrometry

## Abstract

This study aimed to prepare a molecularly imprinted monolithic extraction column (MIMC) inside a micropipette tip by situ polymerization with roxithromycin as the dummy template. The polymers possessed excellent adsorption capacity and class-specificity to multiple macrolide drugs. MIMC was directly connected to a syringe for template removal and for the optimization of extraction conditions without any other post-treatment of polymers. A liquid chromatography-tandem mass spectrometric method was developed for the selective microextraction and determination of macrolide antibiotics in animal muscles based on MIMC. High recoveries of 76.1–92.8% for six macrolides were obtained with relative standard deviations less than 10.4%. MIMC exhibited better retention ability and durability when compared with the traditional C18 and HLB cartridges. The proposed method shows a great potential for the analysis of macrolide drugs at the trace level in animal foodstuffs.

## 1. Introduction

Macrolide antibiotics (MALs), a group of alkalescent antibiotics that are produced by *Streptomycetes*, consist of a large lactone ring (12–16 carbons atoms) and sugar moieties. MALs are used in animal husbandry for therapy and prophylaxis because of their antibacterial activity against gram-positive bacteria and some gram-negative cocci. MALs have been extensively introduced as feed supplements in industrialized animal production since the discovery that antibiotics could increase animal growth rate in 1940s [1,2]. Despite their benefits to animal production, the incorrect use of these drugs may leave residues in food products, bringing adverse effects to human health, such as allergic dermatitis [3]. Researchers have confirmed that the increase of drug resistance is associated with the massive consumption of MALs in animal husbandry [4,5]. As a result of increasing concerns over drug resistance, international organizations, including the European Union [6], United States [7], and China [8], have established maximum residue limits for MALs in animal products. Besides, spiramycin (SPM), tylosin (TYL), and tilmicosin (TIM), which were used as animal growth promoters before, are banned or severely restricted [9,10]. Worryingly, MALs, including TYL, TIM, erythromycin (ERY), and roxithromycin (ROX), are still frequently detected in animal foodstuffs due to their wide availability and low cost [11,12,13]. Thus, it is necessary to supervise MALs residues in animal-derived food.

At present, analytical methods for drug residues are mainly based on liquid chromatography-tandem mass spectrometry (LC-MS/MS), since it has the merits of unambiguous identification and accurate quantification. LC-MS/MS has become the preferred approach for the determination of MALs residues in animal tissues, milk, egg, and honey [14,15]. To eliminate co-extracted impurities, various purification strategies that are based on solid-phase extraction (SPE) [16], QuEChERS (Quick Easy Cheap Effective Rugged Safe) [17], and matrix solid-phase dispersion [18] have been reported. Among these methods, SPE is the most commonly used technique for the extraction of MALs at the trace-level [19,20]. However, conventional SPE sorbents with the property of reverse phase retention favor nonspecific interaction, leading to the co-extraction of numerous impurities [21]. As a porous material, molecularly imprinted polymer (MIP) has become an appealing alternative for sample purification due to its high recognition to the template and template analogues. Indeed, MIP has predetermined recognition sites, which are complementary to the template in shape, size, as well as functional groups, allowing for specifically enriching target compounds from complex matrices [22].

Current attempts for the synthesis of MALs-MIP include traditional approaches (bulk, precipitation, or suspension) [23,24,25] and some novel strategies that are based on hollow porous material [26], magnetic particles [27], and multi-walled carbon nanotubes [28]. These MIPs were applied to the selective extraction of MALs in animal tissues [27,29] and honey samples [26] based on molecularly imprinted solid-phase extraction (MISPE), magnetic solid-phase extraction (MSPE), and dispersive solid-phase extraction (DSPE). However, traditional MIPs need to be ground, sieved, and sometimes stabilizer is necessary in polymerization, which is time-consuming. Moreover, template leakage and the poor mass transfer of traditional MIP have limited its application. Although surface and hollow porous MIP can solve such problems to some extent, the absence of recognition sites in the solid core of surface MIP can reduce adsorption capacity and hollow porous MIP is inadequate toughness. Keeping these comments in mind, the molecularly imprinted monolithic column (MIMC) prepared by situ polymerization inside a stainless-steel, capillary tube, or micropipette tip is popular for improving adsorption capacity and simultaneously reducing tedious steps without the great loss of polymers [30,31,32]. MIMC directly connected with a syringe has been proved to be a simple and efficient way for microextraction [33,34], known as molecularly imprinted polymer monolith microextraction (MIPMME). To date, only two studies have reported the preparation of MIMC for the selective microextraction of macrolides from human blood plasma [35] and swine muscle [36]. Each study focused on the selective extraction of a single macrolide antibiotic. In addition, the specificity and adsorption differences between the MIMC and non-molecularly imprinted monolithic column (NIMC) have not yet been proven.

This study describes the synthesis of MIMC while using ROX as the virtual template for the selective microextraction of multiple macrolide antibiotics. Synthesis conditions, including monomer, porogen, cross-linker, and initiator were screened and optimized. The adsorption ability and selectivity of MIMC were investigated in more detail, and the toughness of MIMC was demonstrated by repeating several experimental cycles. Further, the microextraction procedures of MIMC were optimized, and finally a LC-MS/MS method that was based on MIPMME was successfully established for the analysis of MAL residues in pork, chicken, and beef samples.

## 2. Materials and Methods

### 2.1. Reagents and Materials

Raw material of ROX (98.5% purity) was obtained from Hubei Prosperity Galaxy Chemical Co., Ltd. (Wuhan, China). Methacryclic acid (MAA, 99% purity), acrylamide (AM, 98.5% purity), and 2,2′-Azobisisobutyronitrile (AIBN, 98% purity) were purchased from Kermel Chemical Reagents Development Center (Tianjin, China). 4-vinyl pyridine (4-VP, 96% purity), 2-hydroxyethyl methacrylate (HEMA, 97% purity), and ethylene glycol dimethacrylate (EGDMA, 98% purity) were obtained from Sigma-Aldrich (St. Louis, MO, USA). The inhibitors inside MAA, 4-VP, and EGDMA were removed by active carbon before use. 

Chromatographic grade reagents: methanol (MeOH), acetonitrile (ACN), and formic acid (FA) were purchased from Fisher Scientific (Fairlawn, NJ, USA). Guangzhou Chemical Reagent Factory (Guangzhou, China) supplied other reagents (analytical grade), including toluene, dodecanol, acetone, and ammonium hydroxide (AM). Oasis HLB (60 mg, 3 mL) and C18 cartridges (60 mg, 3 mL) were purchased from Waters Co. (Milford, MA, USA) and Agilent Technologies Co. (Santa Clara, CA, USA), respectively. A Milli-Q water system (Molsheim, France) was used to produce de-ionized water.

The standards of ERY, TIM, and azithromycin (AZI) were bought from Sigma Chemicals Co. (St. Louis). ROX, SPM, clarithromycin (CLA), and tulathromycin (TUL) were purchased from European Pharmacopoeia (EDQM, Strasbourg, France). The chemical structures of these compounds are shown in Figure S1. The purity of each standard was above 95.5%. The individual stock standard solution (1000 mg/L) was prepared by weighing 10 mg of each standard into a 10 mL of volumetric flask and dissolving with MeOH. The stock solutions were stored at −20 °C and can be kept stable for six months. Intermediate standard solutions (200 mg/L, 100 mg/L, and 10 mg/L) were prepared by diluting stock solutions with MeOH. Mixed working solutions were prepared daily by mixing the intermediate standard solutions and appropriately diluting with MeOH.

### 2.2. Preparation of MIMC

**Figure 1:**
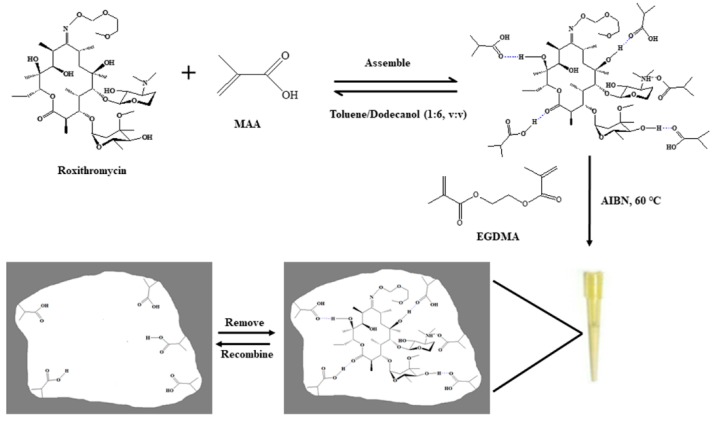
Schematic diagram of the preparation of molecularly imprinted monolithic column.

Figure 1 illustrates the preparation procedure of MIMC. 1 mmoL ROX (template) was dissolved with 12.5 mL of toluene/dodecanol solution (1:6, *v*/*v*) in a 50 mL of polypropylene tube and sonicated for 5 min. 4 mmoL MAA (functional monomer) was added and pre-assembled at 4 °C for 4 h. Subsequently, 20 mmol EGDMA (cross-linker) and 30 mg AIBN (initiator) were successively added. Under the protection of nitrogen, 40 μL of the mixture was transferred into a 200 μL micropipette tip, in which the bottom part has been previously sealed, and then the upper part was sealed with a silicon rubber. The polymerization reaction was allowed to perform in a vacuum chamber at 60 °C for 24 h. After polymerization, the silicon rubbers on both ends of the pipette tip were removed to obtain the MIMC. The MIMC was connected to a 5 mL syringe, and the syringe was then installed on a syringe infusion pump (Baoding Longer Precision Pump Co. Ltd., Baoding, China) for the delivery of loading, washing, and eluting solvents. The template was removed by continually loading MeOH-acetic acid (90:10, *v*/*v*) on MIMC at a flow rate of 0.2 mL/min until no ROX could be detected by high performance liquid chromatography with evaporative light-scattering detector (HPLC-ELSD). 20 mL of MeOH was used to remove the remaining acetic acid. NIMC was prepared by the same procedure, but without adding the template.

### 2.3. Equipments of Characterization

A ZEISS EVO18 microscope (Jena, Germany) was used to obtain the scanning electron microscope (SEM) images of MIMC and NIMC. 

A Micromeritics Gemini VII 2390 surface area analyzer (Atlanta, GA, USA) was applied to evaluate the specific surface area via the BET method. 

Fourier-transform infrared spectroscopy (FT-IR) was carried out by a Thermo Nicolet 6700 Fourier transform infrared spectrometer (Thermo Nicolet, Waltham, MA, USA) with anhydrous KBr as the background. The IR spectra were recorded from 500 to 4000 cm^−1^.

Thermostability of MIMC was determined by a PerkinElmer STA 6000 thermo gravimetric analyzer (PerkinElmer, Foster City, CA, USA). Analysis was performed at the following parameters: nitrogen pressure, 0.2 mPa; initial temperature, 50 °C; final temperature, 600 °C; heating rate, 30 °C/min.

### 2.4. Binding Assays

The polymer particles of MIMC and NIMC were collected and dried before use. The adsorption capacities for single and multiple macrolide drugs (ERY, ROX, CLA, AZI, TUL, TIM, and SPM) were evaluated. 20.0 mg of polymers were incubated with 5 mL of single/mixed standard solutions in ACN (200 mg/L) at 25 °C for 24 h. After centrifugation at 15000 rpm for 5 min., the free MALs in the supernatant were detected by HPLC-ELSD. The adsorption amounts of MIMC and NIMC were calculated by the equation:

$$Q=\left(C_{0}-C_{e}\right)\times v∕m,$$

where *Q* (mg/g) is the adsorption amounts at equilibrium; *C*_0_ (mg/L) and *C_e_* (mg/L) are the initial and equilibrium concentration, respectively; *v* (L) is the volume of standard solution; and, *m* (mg) is the weight of polymers. Imprinting factor (*IF*) was introduced to evaluate the specific recognition ability according to the equation:

$$IF=Q_{MIMC}∕Q_{NIMC},$$

where *Q_MIMC_* and *Q_NIMC_* (mg/g) are the adsorption amounts of MIMC and NIMC, respectively. 

### 2.5. Sample Preparation

Chicken, pork, and beef were purchased from local supermarkets (Guangzhou, China) and there were no MALs residues in these samples through a previous LC-MS/MS analysis. 2 g homogenized samples were weighed into 15 mL polypropylene centrifuge tubes. For recovery test, 100 μL of mixed solutions was added into the muscle samples to prepare three spiked levels (5.0, 10, and 25 μg/kg). The spiked samples were kept at room temperature for 30 min. before proceeding. 

5 mL of ACN was used to extract MALs with the help of ultrasonic and shaking extraction for 10 min. After centrifugation at 8000 rpm for 5 min., the supernatant was transferred into a new centrifuge tube. Another 5 mL of ACN was added into the residue to repeat the extraction. The extract solutions were combined for cleanup.

1 mL of ACN was applied to condition MIMC, and then 5 mL of extract solution was loaded on MIMC at the rate of 0.2 mL/min. MIMC was successively rinsed with 1 mL of ACN and 1 mL of water. 1 mL of 2% ammonium hydroxide in MeOH was used to elute the target compounds. The eluate was dried under a gentle stream of nitrogen at 40 °C, and finally, the residues were reconstituted with 1 mL of 20% MeOH in water solution (containing 0.1% formic acid) for LC-MS/MS analysis. 

### 2.6. HPLC and LC-MS/MS Analysis

The binding assays and optimization of MIPMME procedures were performed on HPLC-ELSD, according to our previous study [37]. 

LC–MS/MS was applied for the recovery and reusability test of MIMC. LC–MS/MS conditions, including mass parameters, mobile phase and separation program, were the same as the reported method [29]. Briefly, an Agilent 1200 HPLC system (PaloAlto, CA, USA) and an Agilent Zorbax SB-Aq C18 column (150 mm × 2.1 mm i.d., 3.5 µm) separated the MALs. The mobile phase consisted of ACN (solvent A) and 0.1% FA in water (solvent B) with the following gradient elution: 0.0–5.0 min. 10–60% A; 5.0–7.0 min. 60–45% A; 7.0–7.01 min. 45–10% A; 7.01–15 min. 10% A. The flow rate was 0.25 mL/min and the injection volume was 10 µL. Mass analyses were performed on an Applied Biosystems API 4000 triple quadrupole mass spectrometer (Foster City, CA, USA) under the positive electrospray ionization mode. Table S1 gives the mass parameter of each analyte.

## 3. Results and Discussion

### 3.1. Preparation of MIMC

**Figure 2:**
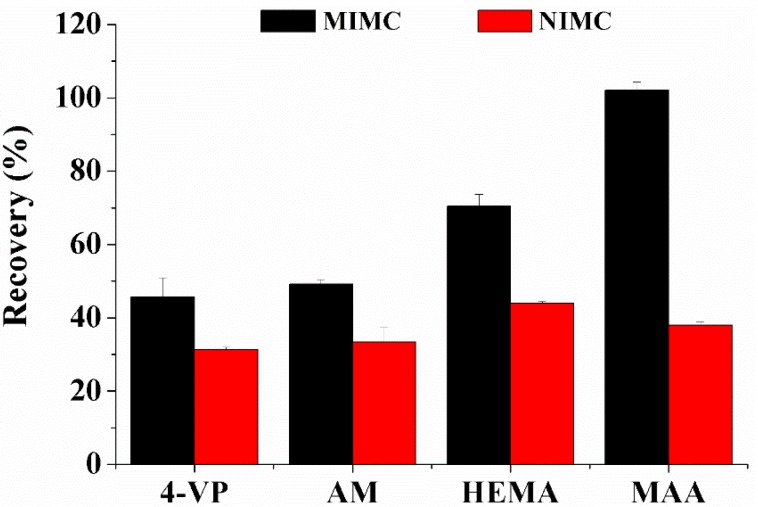
Effect of molecularly imprinted monolithic column prepared by different functional monomers on the recovery of roxithromycin.

The volume and type of monomer and porogen are the two important factors which can impact the structural, physical, and molecular recognition properties of polymers [38], so four types of functional monomers (MAA, AM, HEMA, and 4-VP) were estimated. 1 mL of ROX in ACN (10 μg/mL) was percolated through the MIMC/NIMC and the analysis was performed by HPLC-ELSD. Figure 2 gives the results. Fairly high recovery of ROX (more than 95%) was obtained from MIMC, with MAA as the functional monomer, which was more than twice that of NIMC, which suggested the high specificity of MIMC. Although HEMA provided satisfactory retention for ROX (recovery above 70%) on MIMC, HEMA was not selected for further optimization, because of the IF value less than 1.5. MIMC failed to form rigid polymers with the use of 4-VP (an alkaline monomer), leading to the poor retention of ROX (less than 50%). As a hydrogen bond donator, the carboxyl group of MAA was more likely to participate in the formation of hydrogen bonds with abundant hydroxyl groups of the template. As previous studies discovered [25,26,27], with the 4:1 ratio of MAA to template, the polymers gained high adsorption capacity and specificity to MALs. Based on this ratio, other polymerization parameters were investigated while using single factor analysis. 

Generally, the combination of toluene and dodecanol as porogen can produce porous structures in situ polymerization, which might be beneficial to the permeability of MIMC [36]. Different ratios of toluene to dodecanol (1:4, 1:5, 1:6, 1:7, and 1:8, *v*/*v*) as porogen candidates were evaluated. As shown in Figure S2A, the recovery of ROX in MIMC significantly reduced when the dodecanol percentage was higher than 1:6, while, ROX had the different destiny in NIMC. This trend could be explained by the fact that the higher ratio of dodecanol in porogen resulted in the formation of soft and low specific polymers, and thus ROX did not retain well in these polymers. Although the percentage of toluene-dodecanol (1:4, *v*/*v*) provided satisfactory recovery, as well as specificity, the loading process has been prolonged, owing to the decrease of the permeability of MIMC. To make a compromise, toluene-dodecanol (1:6, *v*/*v*) was deemed as the most suitable porogen. From Figure S2B, it was obvious that the increasing porogen volume did not favor the retention of ROX in MIMC. However, when the volume of porogen was less than 12.5 mL, the polymers were highly rigid, which made the following MIPMME step cumbersome because of the low porosity and permeability. Thus, 12.5 mL of toluene-dodecanol (1:6, *v*/*v*) was used as the porogen for further experiments.

EGDMA was used as cross-linker after the self-assembly of MAA and ROX, followed by the addition of AIBN as the initiator to accelerate polymerization reaction. The amounts of EGDMA and AIBN were estimated to obtain highly specific MIMC. As presented in Figure S2C,D, with the increase of EGDMA and AIBN, there was no significant improvement in the retention of ROX both in MIMC and NIMC. Nevertheless, when EGDMA was less than 15 mmol, the polymers were tightly tiny particles, so that the permeability of MIMC was fairly poor. Besides, it was rather difficult to form rigid-shape polymers with the amount of AIBN less than 20 mg. At the participation of 20 mmol EGDMA and 30 mg AIBN, we successfully synthesized MIMC with excellent performance (favorable permeability, ruggedness, and adsorption capacity).

### 3.2. Characterization of MIMC

**Figure 3:**
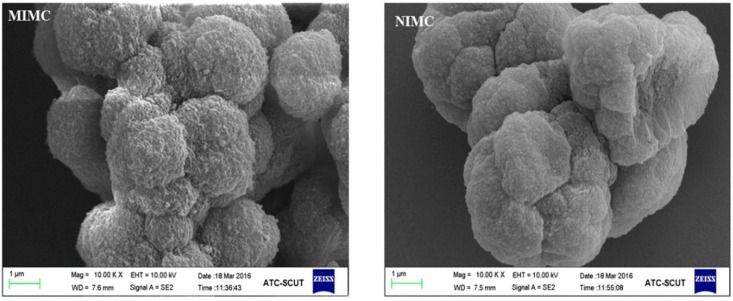
Scanning electron microscope images of molecularly imprinted monolithic column (MIMC) and non-molecularly imprinted monolithic column (NIMC).

Figure 3 gives representative SEM images of MIMC and NIMC. The surface of MIMC was rougher and more porous than that of NIMC, which could improve the mass transfer rate and provide larger surface areas for binding compounds. The specific surface areas that were obtained by using BET model were 191.5 m^2^/g for MIMC and 123.3 m^2^/g for NIMC, thus confirming the presence of more imprinted cavities in MIMC. 

The IR spectra of MIMC and NIMC were similar (Figure S3). Adsorption bands at around 3543 cm^−1^ and 2990 cm^−1^ were the stretching vibrations of O–H and C–H bonds, respectively. An obvious absorption peak at 1731 cm^−1^ was attributed to the stretching vibration of C=O, which was provided by EGDMA and MAA. The weak adsorption peak at 1636 cm^−1^ was assigned to the C=C vibration, which indicated the successful polymerization. 

Thermogravimetric analysis revealed that the decomposition of MIMC occurred at 260 °C and completed at 450 °C (loss of 95% weight), suggesting the outstanding thermal stabilities of MIMC at extreme conditions.

### 3.3. Binding Assays

Adsorption capacities of MIMC and NIMC to single and multiple macrolide drugs (spiked concentration: 200 mg/L of each compound in ACN) were investigated. 5 mL of ACN as a blank reference was subjected to the same procedure to ensure no template-bleeding in the whole process. As listed in Table 1, there was a great difference between the adsorption amounts of MIMC and NIMC, resulting from the abundant binding sites in MIMC. The specificity of MIMC seemed to be related to the number of carbons atoms within the lactone ring, that is, the higher specificity (*IF* > 2.0) of MIMC showed to 15-membered ring macrolides (ROX, CLA, and ERY), meaning higher affinity of MIMC to analogues whose molecular structures are highly similar to the template. Furthermore, MIMC could well recognize multiple macrolides and the *IF* values were higher than 1.5, to some extent, revealing the class-specificity. When compared with single analyte, there was competitive adsorption among multiple macrolides due to the limited binding sites that presented in MIMC. MIMC showed high specific adsorption to CLA and ERY (*IF* > 2.0), because their ring sizes and spatial arrangement of glycosidic side chains are highly similar to ROX, so they could quickly occupy the imprinted cavities that were created by the template [29]. These results indicated that not only lactone ring sizes of MALs, but the space structure of glycosidic side chains will play an important role in specific recognition of MIMC.

### 3.4. Optimization of MIPMME Procedure

#### 3.4.1. Packing Volume

The binding capacity of MIMC to MALs heavily relies on the amounts of polymers. Generally, the more polymers that we added, the more MALs could be well retained on MIMC. At the same time, more polymers will prolong the loading time, which is time-consuming and can reduce the microextraction efficiency. Consequently, the volume of polymerization system packed inside micropipette tip was measured. As presented in Figure S4, the recovery of ROX from MIMC rapidly increased with the rising of polymerization volume and then gradually reached the equilibrium when the volume was up to 40 μL. Meanwhile, NIMC provided steadily growing recovery to ROX, which suggested the enhancement of non-specificity. Thus, 40 μL of polymerization mixture was packed into micropipette tips to synthesize the MIMC.

#### 3.4.2. Loading Solvent

**Figure 4:**
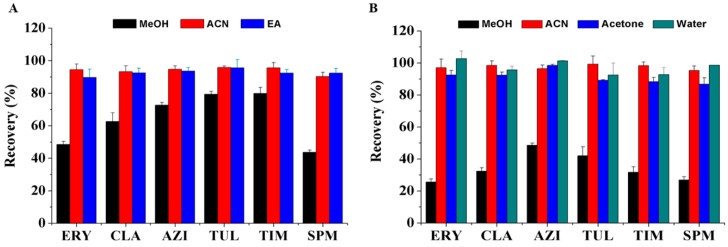
Effects of (**A**) methanol (MeOH), acetonitrile (ACN) and ethyl acetate (EA) as loading solvents and (**B**) MeOH, ACN, acetone and water as washing solvents on the recoveries of six macrolide drugs: erythromycin (ERY); clarithromycin (CLA); azithromycin (AZI); tulathromycin (TUL); tilmicosin (TIM); and, spiramycin (SPM).

The molecular recognition of MIPs is mainly based on hydrogen bonding interactions between the MIPs and analytes, and such interactions are often more stable in weak polar media [39]. Organic solvents with various polarities, including MeOH, ACN, and ethyl acetate (EA), which are commonly used to extract MALs from animal tissues, were investigated as the loading candidates (fortified concentration: 10 μg/mL of six macrolides in 5 mL of each candidate solution). While considering the possible leakage of template during LC-MS/MS analysis, ROX was not estimated in the following programs. Figure 4A gives the results. Satisfactory recoveries of target compounds (above 95%) were obtained while using ACN and EA as the loading solvents. In sharp contrast, MeOH provided low recoveries for six MALs, especially for SPM (less than 50%), which might be caused by the strong suppression of high polar solvent (MeOH) to imprinted interaction. We selected ACN as the loading solvent for further study in view of easy volatilization of EA and consistency with the extraction solvent. 

#### 3.4.3. Washing Solution

The optimization of washing solution is a crucial step for imprinted extraction in eliminating the co-extracted impurities with less loss of analytes and to simultaneously reduce the non-specific adsorption. Several solvents with different polarity (MeOH, ACN, acetone, and water) as washing solutions were estimated after loading with extract solution. As shown in Figure 4B, MeOH had washed off lots of analytes and the recoveries were below 50%. It has been reported that polar solvents, such as MeOH, could disrupt the non-covalent interactions between analytes and MIPs [40]. On the contrary, there was no obvious loss of six MALs for MIMC washing with ACN, acetone, and water. However, the recoveries were also high on NIMC (above 80%) when acetone and water were used as the washing solvents. Ultimately, the MIMC was successively rinsed with ACN and water, which could effectively remove the lipid and water-soluble impurities. Under optimal conditions, non-specificity adsorption could be reduced, whereas most of the analytes were trapped in the polymer because of the specific interactions. The recoveries of six MALs on MIMC were more than twice that on NIMC.

#### 3.4.4. Eluting Solution

MeOH and different percentages of ammonium hydroxide in MeOH (1%, 2%, 3%, and 4%) as eluting candidates were assessed in this study in view of easy volatilization of EA and consistency with the extraction solvent. The results (Figure S5) revealed that the participation of AM in eluting solution tended to improve the elution ability, perhaps because of the weak alkalinity of MALs. A higher concentration of AM (>3%) in MeOH did not significantly improve the recoveries of analytes, but it extended the evaporation time. Therefore, 1 mL of 2% ammonium hydroxide in MeOH was chosen for eluting the MALs from MIMC, and the recoveries were higher than 94.5%.

#### 3.4.5. Class-Specificity of MIMC

**Figure 5:**
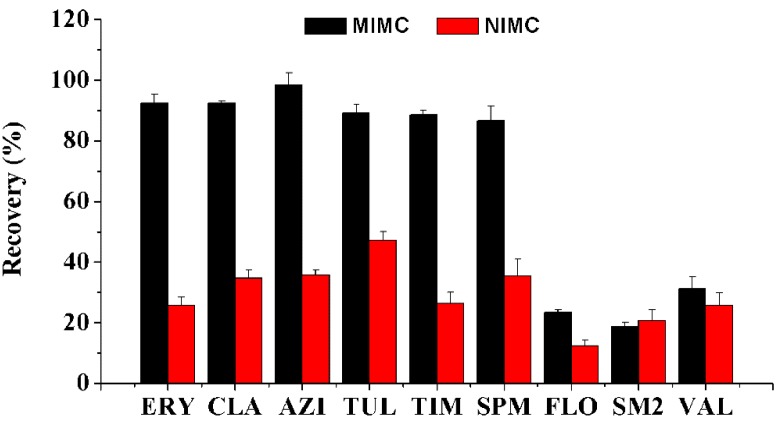
Class-specific adsorption capacities of MIMC and NIMC for six macrolides (same as Figure 4), florfenicol (FLO), sulfadimidine (SM2), and valnemulin (VAL).

The class-specificity of MIMC was investigated through analyzing six MALs and other pharmaceuticals with a large consumption in animal husbandry, including florfenicol (FLO), sulfadimidine (SM2), and valnemulin (VAL). 1 mL of mixed standard solution in ACN (10 μg/mL) was loaded onto MIMC or NIMC, followed by the optimal MIPMME procedure. As illustrated in Figure 5, six MALs were well recognized by MIMC, with their recoveries being higher than 88% due to the complementation between target MALs and imprinted cavities, while poor recoveries were obtained from NIMC. Both MIMC and NIMC presented low affinity to FLO, SM2, and VAL, since their molecular structures are quite different from the template (Figure S1 for their structures). It can be demonstrated that the MIMC had good class-specificity for multiple MALs and it had great potential for the selective separation of MALs.

### 3.5. Comparison of Different Cleanup Methods

The recoveries of six MALs obtained from different SPE cartridges, including MIPMMC, C18, and Oasis HLB, were compared. 1 mL of blank pork matrix was spiked at 10 ng/mL of six MALs for the further SPE procedures. For the C18 cartridge, 3 mL of MeOH and 3 mL of water were used to condition the cartridge. 1 mL of the extract was diluted with 4 mL of water before loading. The C18 cartridge was washed with 3 mL of 10% MeOH in water and the analytes were eluted with 5 mL of 5% ammonium hydroxide in MeOH. For Oasis HLB cartridge, 1 mL of the extract was evaporated and the residues were re-dissolved with 5 mL of phosphate buffer solution (0.1 M, pH = 8.0) before loading. Except for the loading process, cleanup and elution were the same as the C18 cartridge. As shown in Figure S6, the MIMC provided higher recoveries than other cartridges for six MALs, especially for ERY and SPM. It certified that MIMC had excellent purification and enrichment ability to six MALs in animal muscles. 

### 3.6. Reusability of MIMC

The MIMC was employed to repeat several binding and eluting SPE cycles to investigate its reusability (each SEP cycle: 5 mL of pork matrix spiked at 10 ng/mL of six MALs). After each cycle, MIMC was rinsed with 1 mL of 5% ammonium hydroxide in MeOH to remove the residual MALs, and then regenerated by washing with 1 mL of water and 1 mL of MeOH three times. The results exhibited that MIMC could be reused at least 20 times, with only a slight decrease of its recognition properties (recovery loss less than 5%). In contrast, the recoveries for most MALs in C18 cartridge dramatically declined (below 60%) after five cycles. It was obvious that the MIMC had favorable durability, which would be economical and stable in the analysis of real samples.

### 3.7. Application of MIMC in Animal Foodstuff

The developed MIPMME procedure, coupled with LC-MS/MS, was applied to selectively enrich and detect six MALs from chicken, pork, and beef samples. Good linearity was achieved in the concentration range of 1.0–100 μg/kg for target compounds, with correlation coefficients (*r*^2^) higher than 0.99 under the optimized conditions of sample separation and detection (Table 2). Linear equations for target analytes in three matrices are listed in Table S2. The limit of detection (LOD) and limit of quantification (LOQ) were in the range of 0.5–1.0 μg/kg and 2.0–5.0 μg/kg, respectively. 

**Figure 6:**
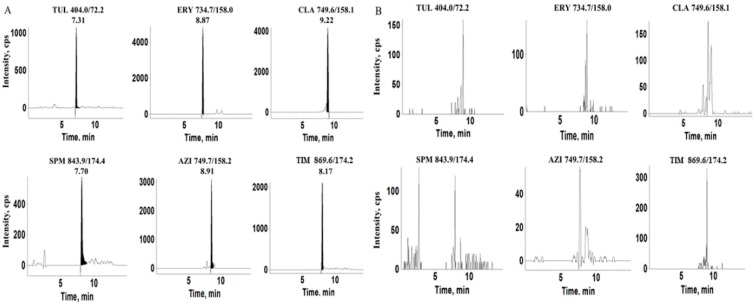
Typical SRM chromatograms obtained from (**A**) spiked chicken matrix at the concentration of 5 μg/kg and (**B**) blank chicken matrix.

Chicken, pork, and beef samples at three spiked concentration levels of six MALs (5.0, 10, and 25 μg/kg) were analyzed to investigate the accuracy and precision of this method. The precision was described as the relative standard deviation (RSD). Table 2 also gives the results. The average recoveries of six MALs in three muscle samples were from 76.1% to 92.8%, with intra-day and inter-day RSDs that were lower than 10.4%. Figure 6 shows the typical SRM chromatograms of spiked chicken sample. Figure S7 shows the chromatograms of spiked pork and beef samples after the MISPE procedure.

Table 3 lists the comparison of other reported methods with the proposed method for the determination of MALs. The LODs of the developed method were lower than those of LC-MS/MS analysis based on traditional extraction approaches, such as pressurized liquid extraction (PLE) [41] and accelerated solvent extraction (ASE) [12]. When compared with novel extraction strategies using MIP technology, the method had lower LODs than the HPLC methods based on multi-walled carbon nanotubes MISPE (MWNTs-MIPSE) [28] and magnetic MISPE (MMISPE) [27]. Two papers described the determination of multiple macrolides in animal foodstuffs by LC-MS/MS based on MISPE [29] and hollow porous MIP-DSPE (HPMIP-DSPE) [26]. Although the LODs of the method that was developed were slightly higher than those of the MISPE method, the recoveries of this method were much better than the latter. The HPMIP-DSPE method had higher recoveries and lower LODs for the analysis of MALs in honey due to the differences between sample matrices. Thus, the LC-MS/MS coupled with MIPMME was efficient and sensitive in analyzing trace amounts of MALs in animal-derived food.

## 4. Conclusions

In this work, the MIP monolithic extraction column for MALs was prepared inside a micropipette tip while using ROX as the dummy template. MIMC was directly connected with a syringe to perform template removal and the following microextraction procedures, which is simple and convenient for sample pre-treatment. Based on MIMC, a LC-MS/MS method was established for the analysis of MALs in animal muscle samples. The developed method provided good linearity, high sensitivity, and satisfactory recoveries for MALs within the experimental concentration ranges. When compared with traditional C18 and HLB cartridges, MIMC showed more favorable retention ability for six MALs and it can be reused at least 20 times. Thus, the proposed method was selective, efficient, and economical for the monitoring of trace MALs residues in animal foodstuffs. 

## Figures and Tables

**Table 1:** Specific adsorption capacities of MIMC and NIMC for single and multiple macrolides *^a^*.

Compound	Polymer *^b^*	Single		Multiple	
		Q (mg/g) *^c^*	*IF ^d^*	Q (mg/g)	*IF*
Roxithromycin	MIMC	12.0	2.8	3.4	1.7
	NIMC	4.3		1.9	
Clarithromycin	MIMC	11.5	2.6	3.5	2.3
	NIMC	4.5		1.5	
Erythromycin	MIMC	11.5	2.3	8.2	2.1
	NIMC	5.0		3.7	
Azithromycin	MIMC	12.4	1.7	3.4	1.8
	NIMC	7.2		1.9	
Tulathromycin	MIMC	25.2	1.6	18.0	1.4
	NIMC	18.4		12.9	
Tilmicosin	MIMC	17.1	1.8	5.1	1.6
	NIMC	9.3		3.2	
Spiramycin	MIMC	10.2	2.0	4.8	1.8
	NIMC	5.1		2.6	

*^a^* multiple macrolides (roxithromycin, clarithromycin, erythromycin, azithromycin, tulathromycin, tilmicosin and spiramycin), 200 mg/L of each drug in acetonitrile; *^b^* MIMC, molecularly imprinted monolithic column; NIMC, non-molecularly imprinted monolithic column; *^c^* Q, adsorption capacity; *^d^ IF*, imprinting factor.

**Table 2:** Validation data for six macrolide antibiotics after molecularly imprinted polymer monolith microextraction (MIPMME) procedure in spiked animal muscle samples *^a^*.

Compound	Samples	Linearity (r^2^)	LOD *^b^* (μg/kg)	LOQ *^c^* (μg/kg)	Intra-Day Recovery (%RSD, *n* = 6) *^d^*			Inter-Day Recovery (%RSD, *n* = 18)		
					5 μg/kg	10 μg/kg	25 μg/kg	5 μg/kg	10 μg/kg	25 μg/kg
Erythromycin	chicken	0.9977	0.5	2.0	80.4(4.2)	82.8(2.7)	85.4(1.7)	81.2(5.1)	83.3(2.4)	86.1(2.5)
	pork	0.9984	0.5	2.0	81.7(3.8)	83.4(3.1)	84.8(5.9)	82.7(4.4)	83.9(3.6)	85.6(6.7)
	beef	0.9962	0.5	2.0	76.8(7.2)	78.6(3.4)	82.1(4.9)	78.2(10.4)	79.1(5.6)	83.7(7.1)
Clarithromycin	chicken	0.9995	0.5	2.0	84.9(6.3)	85.7(2.1)	88.4(1.6)	85.3(8.2)	84.9(3.6)	89.7(2.4)
	pork	0.9992	0.5	2.0	82.6(8.1)	84.7(1.8)	85.1(4.7)	80.2(9.8)	84.1(1.1)	86.4(5.3)
	beef	0.9989	0.5	2.0	80.5(4.7)	82.6(0.7)	84.6(4.2)	82.9(5.1)	83.7(2.6)	85.1(3.9)
Tulathromycin	chicken	0.9992	1.0	5.0	75.8(5.8)	78.1(3.4)	79.1(2.3)	76.1(3.7)	78.6(1.9)	80.3(2.7)
	pork	0.9994	1.0	5.0	76.8(1.2)	78.8(6.1)	80.2(1.9)	77.5(1.6)	79.2(3.4)	81.2(2.1)
	beef	0.9988	1.0	5.0	75.3(8.3)	76.7(2.8)	78.3(5.4)	76.9(5.8)	78.1(4.2)	79.8(2.9)
Azithromycin	chicken	0.9963	0.5	2.0	83.5(2.5)	84.8(2.2)	85.7(3.6)	83.9(1.4)	84.4(3.6)	87.4(7.2)
	pork	0.9998	0.5	2.0	85.1(7.3)	88.4(5.4)	91.7(6.7)	86.3(8.1)	89.1(4.5)	92.8(3.1)
	beef	0.9992	0.5	2.0	78.4(5.7)	80.2(6.5)	90.6(5.0)	79.6(6.4)	83.2(8.2)	86.1(4.6)
Spiramycin	chicken	0.9961	1.0	5.0	84.6(3.2)	86.9(1.2)	87.6(1.1)	85.3(5.8)	87.2(2.7)	87.6(4.1)
	pork	0.9987	1.0	5.0	82.3(4.1)	83.4(8.3)	85.8(1.7)	83.5(2.5)	84.8(7.2)	85.7(3.6)
	beef	0.9992	1.0	5.0	79.2(4.6)	81.8(6.7)	83.6(1.5)	78.2(5.7)	83.4(8.9)	84.3(2.1)
Tilmicosin	chicken	0.9930	0.5	2.0	85.1(1.5)	89.2(7.4)	91.8(2.5)	86.5(3.2)	90.6(7.1)	91.2(6.2)
	pork	0.9943	0.5	2.0	82.5(3.4)	86.6(1.9)	90.7(3.6)	81.2(4.5)	87.2(2.5)	89.1(7.8)
	beef	0.9985	0.5	2.0	79.4(1.9)	84.2(6.4)	88.1(2.7)	78.2(2.2)	86.2(8.1)	90.3(4.5)

*^a^* MIPMME, molecularly imprinted polymer monolith microextraction; *^b^* LOD, limit of detection; *^c^* LOQ, limit of quantification; *^d^* RSD, relative standard deviation.

**Table 3:** Comparison of the method developed with other reported methods.

Preparation *^a^*	Analytical Method *^b^*	Matrix	Analyte *^c^*	LOD (μg/kg) *^d^*	Recovery (%)	Ref.
PLE	LC-MS/MS	chicken	SPM, TIM, TYL	1.0–6.0	77.1–94.0	[41]
ASE	LC-MS/MS	pork, kidney, liver	SPM, OLE, ERE, DOR, IVE, TIM, ERY, ROX, CLA, KIT, MED	0.2–0.6	76.0–102	[12]
MWNTs-MISPE	HPLC-UVD	chicken	ERY	-	85.3–95.8	[28]
MMISPE	HPLC-UVD	pork, fish, shrimp	ERY, OLE, AZI, TIM, CLA, ROX	1.5–20	64.8–84.2	[27]
HPMIP-DSPE	LC-MS/MS	honey	AZI, SPM, TIM, CLA, JOS, ROX, TYL	0.003–0.017	88.0–117	[26]
MISPE	LC-MS/MS	pork, beef, chicken	AZI, TUL, SPM, TIM, ERY, CLA, ROX, MED, JOS, KIT	0.1–0.4	60.7–100	[29]
MIPMME	LC-MS/MS	pork, beef, chicken	CLA, ERY, ZAI, TUL, TIM, SPM	0.5–1.0	76.1–92.8	This work

*^a^* PLE, pressurized liquid extraction; ASE, accelerated solvent extraction; MWNTs-MISPE, molecularly imprinted solid-phase extraction based on multi-walled carbon nanotubes; MMISPE, magnetic molecularly imprinted solid-phase extraction; MISPE, molecularly imprinted solid-phase extraction; HPMIP-DSPE, dispersive solid-phase extraction based on hollow porous molecularly imprinted polymers; MIPMME, molecularly imprinted polymer monolith microextraction; *^b^* LC-MS/MS, liquid chromatography-tandem mass spectrometry; HPLC-UVD, high-performance liquid chromatography with ultraviolet detector; *^c^* SPM, spiramycin; OLE, troleandomycin; ERE, erythromycin ethylsuccinate; DOR, doramectin; IVE, ivermectin; TIM, tilmicosin; ERY, erythromycin; ROX, roxithromycin; CLA, clarithromycin; KIT, kitasamycin; MED, midecamycin; JOS, josamycin; AZI, azithromycin; TYL, tylosin; *^d^* LOD, limit of detection; LOQ, limit of quantitation.

## Supplementary Materials

The following are available online at https://www.mdpi.com/2073-4360/11/7/1109/s1: Figure S1, Chemical structures of macrolide antibiotics; Figure S2, Effects of (A) different ratio of toluene to dodecanol as porogen, (B) porogen volume, (C) different amounts of EGDMA as cross-linker and (D) AIBN as initiator on the recovery of roxithromycin obtained from MIMC and NIMC; Figure S3, FT-IR characterization of MIMC and NIMC; Figure S4, Effect of polymerization volume on the recovery of roxithromycin for MIMC and NIMC; Figure S5, Effect of MeOH and different percentages of ammonium hydroxide (AM) in MeOH as elution solutions on the recoveries of macrolides drugs; Figure S6, The comparison of MIMC, C18 and Oasis HLB cartridges on the recoveries of target macrolides (the abbreviations are same as Figure 4) at 10 ng/mL spiked concentration of six macrolides in pork matrix; Figure S7, Typical SRM chromatograms obtained from (A) spiked pork and (B) spiked beef matrices at the concentration of 5 μg/kg and their corresponding blank matrices; Table S1, SRM parameters for target analytes in positive ion mode; Table S2, Linear equation for each analyte in three matrices.
