# Supplementary material for: Preparation and Application of Molecularly Imprinted Monolithic Extraction Column for the Selective Microextraction of Multiple Macrolide Antibiotics from Animal Muscles
Source: Polymers (Basel). 2019 Jul 1;11(7):1109. doi: 10.3390/polym11071109 (PMC6680429; doi:10.3390/polym11071109)
Supplement: Supplementary file 1 [file polymers-11-01109-s001.pdf]

Supplementary information

Preparation and application of molecularly imprinted monolithic extraction column for the selective microextraction of multiple macrolide antibiotics from animal muscles

Xuqin Song [1], Tong Zhou [1], Jiahui Zhang [1], Yijuan Su [2,*], Hao Zhou [1] and Limin He [1,*]

[1] National Reference Laboratory of Veterinary Drug Residues (SCAU), College of Veterinary Medicine, South China Agricultural University, Guangzhou 510642, China; song1991yi@163.com; zhoutong.only@163.com; jiahuiz@scau.edu.cn; zhouhao120928@163.com; liminokhe@scau.edu.cn

[2] Department of Ecology, College of Natural Resources and Environment, South China Agricultural University, Guangzhou, 510642, China; syj@scau.edu.cn

* Correspondence: liminokhe@scau.edu.cn; Tel.: +86-2085284896

* Co-Correspondence: syj@scau.edu.cn; Tel.: +86-2085281887

Received: date; Accepted: date; Published: date

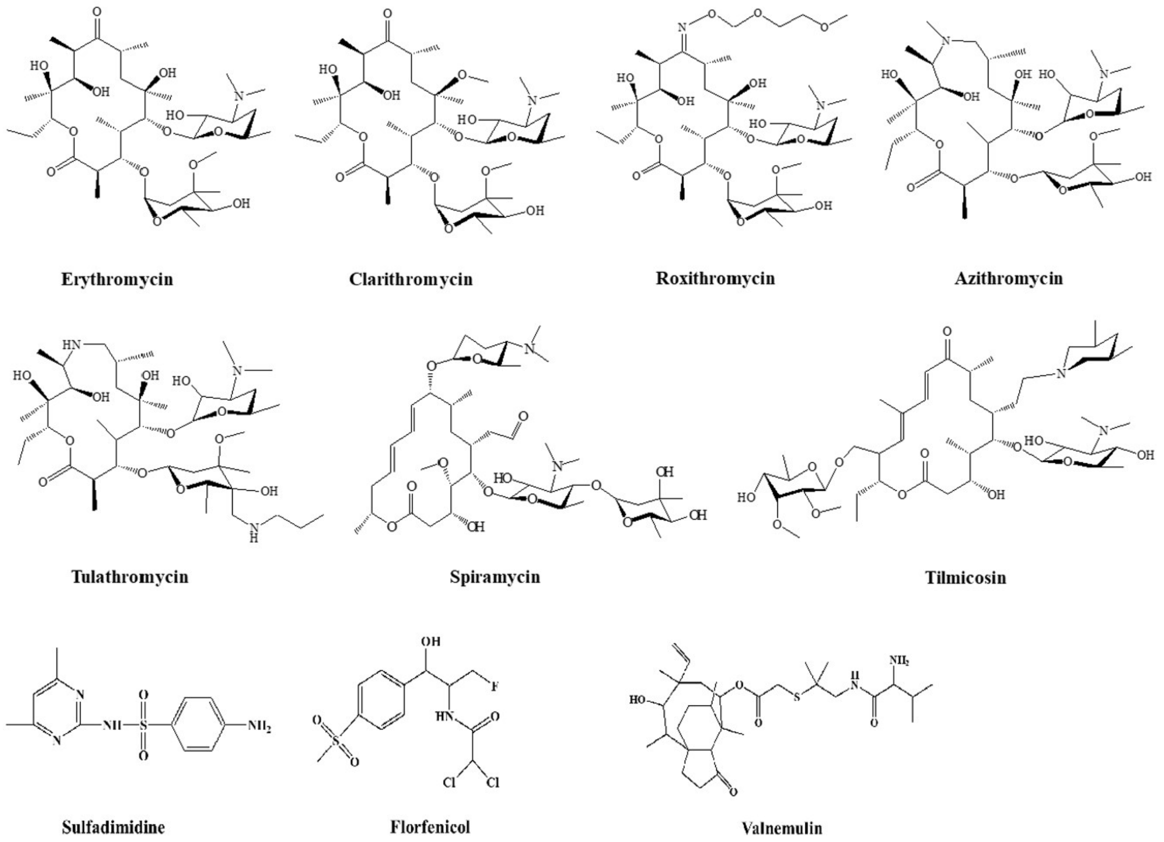

Figure S1. Chemical structures of macrolide antibiotics, florfenicol, sulfadimidine and valnemulin

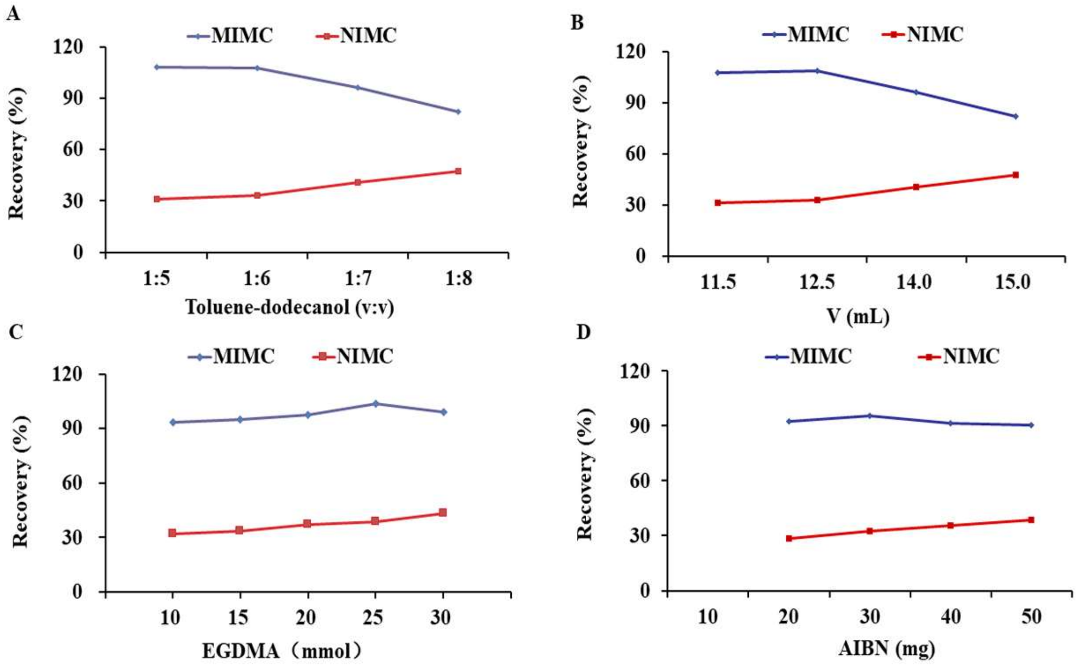

Figure S2. Effects of (A) different ratio of toluene to dodecanol as porogen, (B) porogen volume, (C) different amounts of EGDMA as cross-linker and (D) AIBN as initiator on the recovery of roxithromycin obtained from MIMC and NIMC

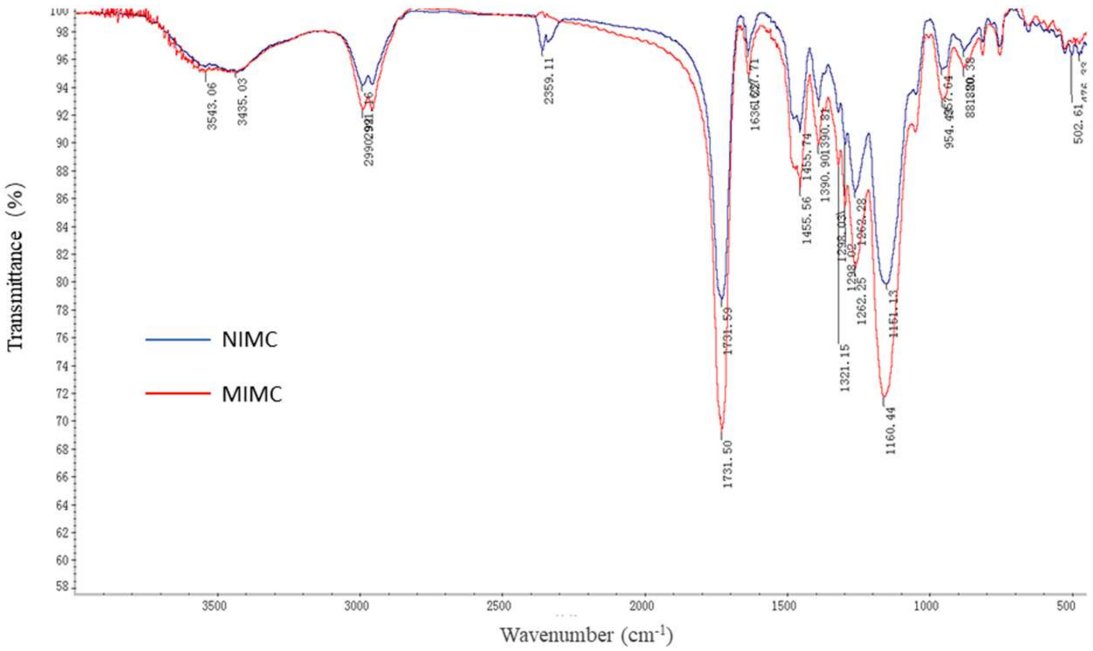

Figure S3. FT-IR characterization of MIMC and NIMC

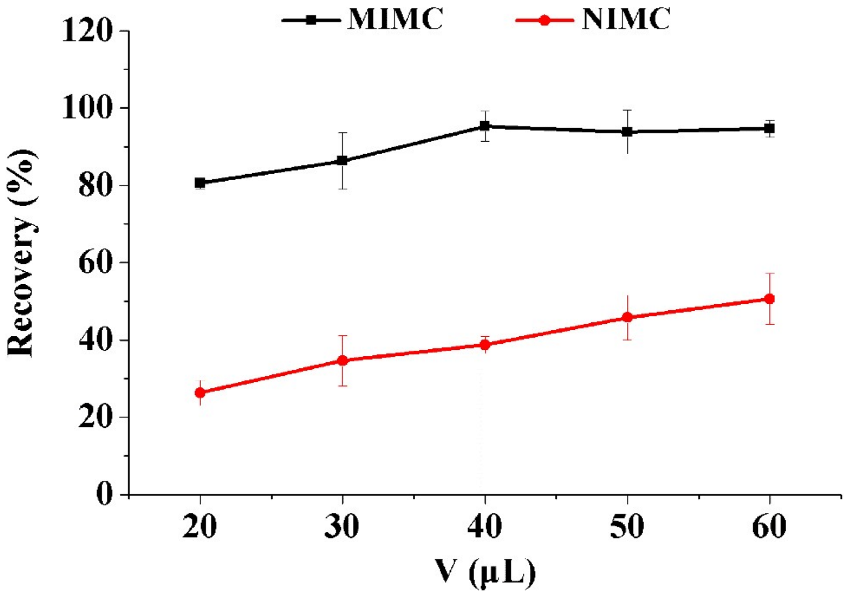

Figure S4. Effect of polymerization volume on the recovery of roxithromycin for MIMC and NIMC

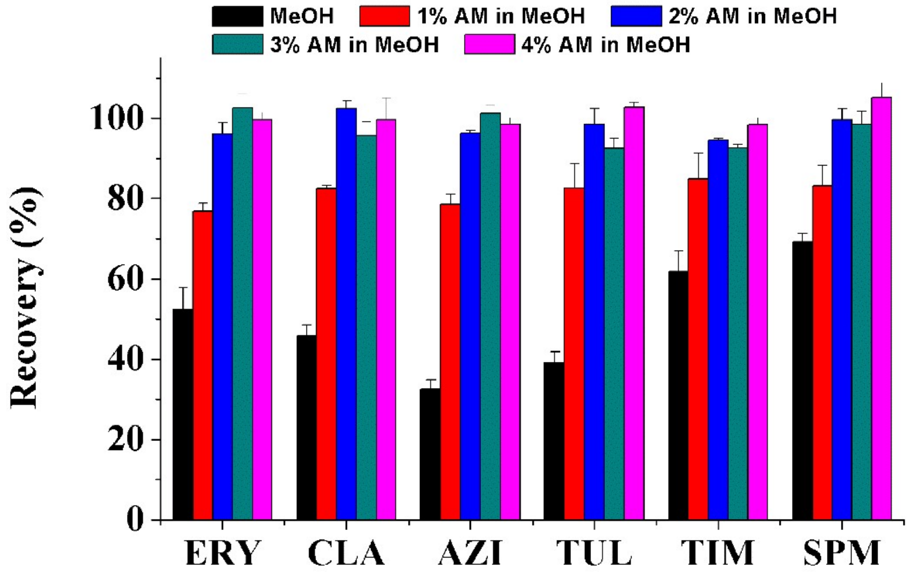

Figure S5. Effect of MeOH and different percentages of ammonium hydroxide (AM) in MeOH as elution solutions on the recoveries of macrolides drugs.

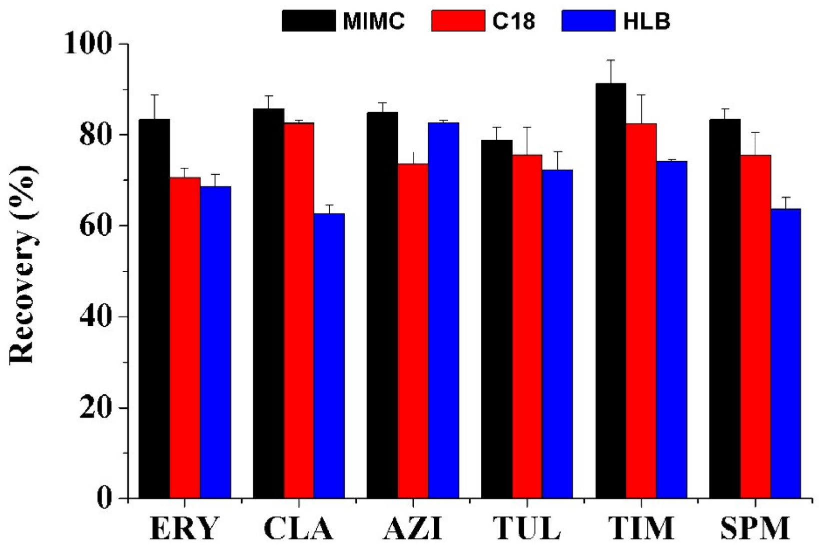

Figure S6. The comparison of MIMC, C18 and Oasis HLB cartridges on the recoveries of target macrolides (the abbreviations are same as Figure 4) at 10 ng/mL spiked concentration of six macrolides in pork matrix

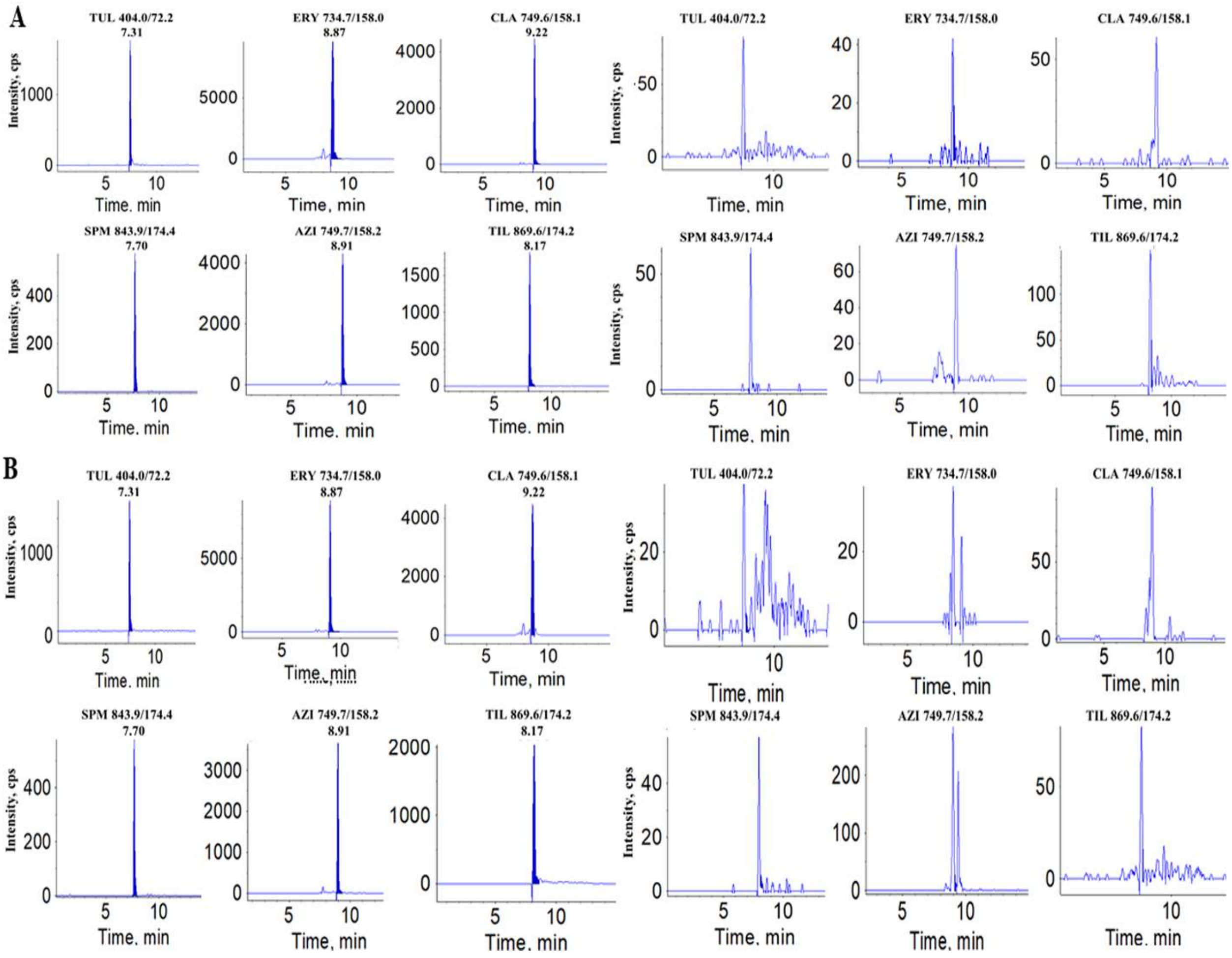

Figure S7. Typical SRM chromatograms obtained from (A) spiked pork and (B) spiked beef matrices at the concentration of 5 μg/kg and their corresponding blank matrices

Table S1. SRM parameters for target analytes in positive ion mode [a]

**Compounds**	**Abbr.** [b]	**Precursor ion [M + H]+**	**Product ion** [c]	**DP** [d] **(V)**	**CE** [e] **(eV)**	**RT** [f] **(min)**
Tilmicosin	TIM	869.6	696.4*	130	60	8.26
			174.2	130	66	
Spiramycin	SPM	843.9	141.9*	110	50	7.85
			174.4	110	48	
Azithromycin	AZI	749.7	591.8*	80	46	9.11
			158.2	75	28	
Clarithromycin	CLA	749.6	591.5*	80	41	9.20
			158.1	80	26	
Erythromycin	ERY	734.7	576.5*	64	43	8.97
			158.0	64	27	
Tulathromycin	TUL	404	158.2*	71	33	7.37
			72.2	71	31	

[a] SRM, selected reaction monitoring; [b] Abbr., abbreviations;

[c] Product ion, the first product ion (*) of each analyte was used for quantification, and the second one was used for identification;

[d] DP, declustering potential; [e] CE, collision energy; [f] RT, retention time.

Table S2. Linear equation for each analyte in three matrices

Compounds	Matrix	Linear equation
Erythromycin	chicken	$y=3.82\times10^3\chi+4.51\times10^2$
	pork	$y=4.13\times10^3\chi+1.62\times10^2$
	beef	$y=3.36\times10^3\chi+8.36\times10^2$
Clarithromycin	chicken	$y=2.03\times10^3\chi+1.98\times10^3$
	pork	$y=3.85\times10^3\chi+4.23\times10^3$
	beef	$y=3.63\times10^3\chi+3.81\times10^3$
Tulathromycin	chicken	$y=4.92\times10^2\chi+1.65\times10^2$
	pork	$y=5.19\times10^2\chi+4.26\times10^2$
	beef	$y=5.42\times10^2\chi+3.67\times10^2$
Azithromycin	chicken	$y=2.86\times10^3\chi+1.16\times10^2$
	pork	$y=3.25\times10^3\chi+7.27\times10^2$
	beef	$y=2.26\times10^3\chi+7.61\times10^2$
Spiramycin	chicken	$y=2.03\times10^2\chi+1.81\times10^2$
	pork	$y=2.36\times10^2\chi+2.53\times10^2$
	beef	$y=3.58\times10^2\chi+1.32\times10^2$
Tilmicosin	chicken	$y=7.83\times10^2\chi+3.35\times10^2$
	pork	$y=5.62\times10^2\chi+4.12\times10^2$
	beef	$y=6.15\times10^2\chi+4.69\times10^2$
